# Hypoxia-inducible factor 1α in Schwann cells promotes peripheral nerve myelination

**DOI:** 10.1016/j.jbc.2025.110433

**Published:** 2025-07-01

**Authors:** Yuka Kobayashi-Ujiie, Shuji Wakatsuki, Yurika Numata-Uematsu, Megumi Shibata, Akihito Harada, Yasuyuki Ohkawa, Nobuhito Goda, Toshiyuki Araki

**Affiliations:** 1 Department of Peripheral Nervous System Research, National Institute of Neuroscience, National Center of Neurology and Psychiatry, Tokyo, Japan; 2 Division of Transcriptomics, Medical Institute of Bioregulation, Kyusyu University, Fukuoka, Japan; 3 Department of Life Sciences and Medical Bioscience, Waseda University School of Advanced Science and Engineering, Tokyo, Japan

## Abstract

Schwann cells are essential for supporting the metabolic activity of neurons and myelination in the peripheral nervous system. While hypoxia is known to influence development in aerobic organisms and has recently been shown to regulate oligodendrocyte differentiation in the central nervous system, its role in Schwann cell function remains less understood. Here we demonstrate that hypoxia-inducible factor 1α (HIF1**α**) in Schwann cells promotes peripheral nerve myelination. HIF1α protein expression is post-transcriptionally regulated and highly induced in myelinating Schwann cells during development and after injury. We also demonstrated that peripheral nerve tissue experiences hypoxic conditions during physiological development and during regeneration following injury. Stabilization or overexpression of HIF1α in Schwann cells promotes myelination in culture. Analysis of HIF1α targets revealed that HIF1α upregulates genes associated with Schwann cell myelination and repair. Furthermore, conditional deletion of HIF1α in Schwann cells results in delayed morphological and functional recovery from peripheral nerve injury. Together, these findings identify HIF1α as a novel regulator of Schwann cell myelination and nerve repair.

Myelin is essential for the rapid propagation of action potentials and proper function of the nervous system (1). In the peripheral nervous system (PNS), Schwann cells are responsible for myelin formation, which gives trophic support and protection from external stimuli to maintain axonal integrity (2). Myelinating Schwann cells are developed from neural crest cells through several stepwise differentiation processes (3). Previous reports have shown the role of key transcription factors, including oct6 and krox20, in the differentiation of myelinating Schwann cells (4, 5, 6, 7). However, the molecular mechanisms that regulate myelination remain unclear.

Gaseous oxygen is critical for all aerobic animals to produce ATP *via* mitochondrial respiration and oxidative phosphorylation. Since hypoxia or oxygen deprivation could occur due to a variety of physiological or pathological conditions, organisms have developed adaptive responses to manage such conditions (8, 9, 10). HIF1 is a heterodimeric transcription factor that plays an integral role in the body's response to low oxygen concentrations. HIF1 consists of a constitutively expressed β-subunit and an oxygen-regulated α-subunit. Under normoxic conditions, specific HIF1α prolyl hydroxylases (PHD) hydroxylate two proline residues (Pro-402 and Pro-564) in the oxygen-dependent degradation (ODD) domain of HIF1α. The von Hippel-Lindau protein E3 ubiquitin ligase complex associates with hydroxylated proline residues and targets HIF1α to proteasomal degradation. On the other hand, under hypoxic conditions, proline hydroxylase is inhibited and HIF1α escapes degradation and serves as a transcription factor in the nucleus to activate adaptive responses against hypoxic conditions (11, 12).

Hypoxia has increasingly been recognized to play an important role in animal physiology and development. It has been previously reported that the differentiation of oligodendrocyte progenitors is controlled by HIF1 (13). HIF1 activity in oligodendrocyte progenitors inhibits myelination by inducing autocrine Wnt7a/7b signaling, which also promotes blood vessel growth, suggesting that stem cell niches in the developing embryo brain experience a hypoxic environment. Previous reports using a hypoxia marker, pimonidazole hydrochloride, have shown that hypoxic regions can be detected in different stages of animal development (14, 15), which prompted us to investigate whether HIF1 might have a role in peripheral nerve differentiation as well.

Here, we demonstrate that HIF1 serves as a myelination-promoting transcription factor in Schwann cells and promotes peripheral myelination. We found that HIF1α expression is induced in peripheral nerve Schwann cells in developing nerve and after injury. HIF1α transcriptionally activates myelin genes, including *mbp*. Conditional deletion of HIF1α in Schwann cells impairs remyelination and delays the functional recovery in regenerating nerves after injury. We also found that administration of a pharmacological HIF1α stabilizer increased the number of remyelinating axons in regenerating peripheral mouse nerve. Collectively, our findings reveal that HIF1α is a novel myelination regulator of Schwann cells.

## Results

### HIF1**α** expression is upregulated in Schwann cells during myelination

**Figure 1:**
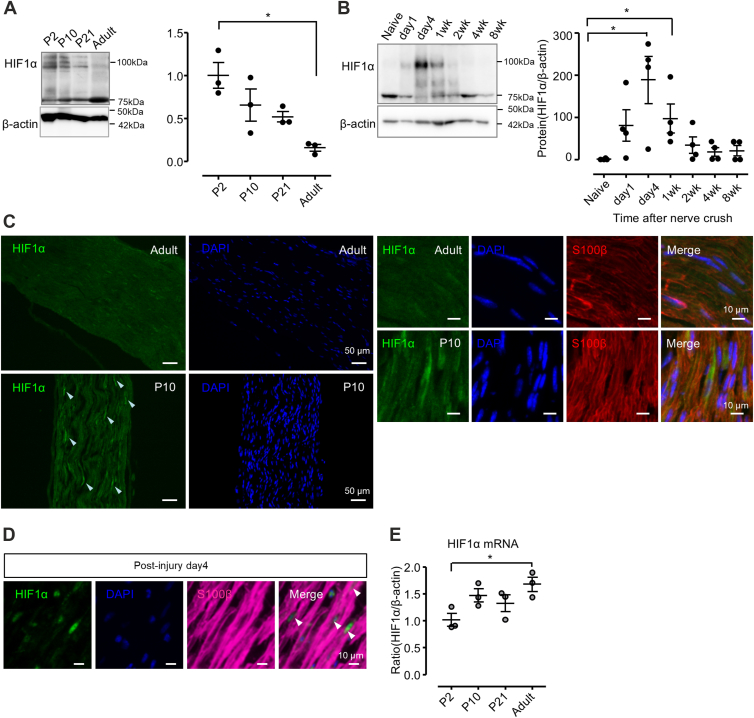
**HIF1α expression is post-transcriptionally regulated in Schwann cells in peripheral nerve during development and after injury**. *A*, representative immunoblot image (*left top*) and quantified expression levels (*right*) of HIF1α protein at indicated time-points in mouse sciatic nerve during development. Quantified data are normalized to and shown as ratio to the expression level of β-actin (*left bottom*). (n = 3; ∗∗*p* ˂ 0.01 and ∗*p* ˂ 0.05 by unpaired Student’s *t* test; mean ± SEM). *B*, representative immunoblot image (*left middle*) and quantified expression levels (*right*) of HIF1α protein at indicated time-points after crush injury in the mouse sciatic nerve segment distal to the injury site. Quantified data are normalized to and shown relative to that of β-actin (*left bottom*). (n = 4; ∗*p* ˂ 0.05 by unpaired Student’s *t* test; mean ± SEM). *C* and *D*, representative photomicrographs showing immunohistochemical localization of HIF1α protein in adult (*A*, *B*, *E*–*H*) and P10 (c,d i-l) mouse sciatic nerve (*C*) and in injured mouse sciatic nerve (4days after injury, *D*). In *C*, *left four panels* show nuclear localization of HIF1α protein (a, c) by co-localization with DAPI (b, d) in low magnification view; and *right eight panels* show its Schwann cell nuclear localization by co-localization with DAPI (*F* and *J*) and S100β (*G* and *K*) in high magnification view. *White arrowheads* in (*C*) indicate HIF1α-positive cells. (Scale bar; 50 μm in a-d, 10 μm in e-l) In (*D*), Schwann cell nuclear localization of HIF1α protein is shown by co-localization with DAPI and S100β. Arrowheads indicate HIF1α-positive Schwann cells. (Scale bar; 10 μm). *E*, HIF1α mRNA expression levels in mouse sciatic nerve during development and in adulthood are shown as ratio to expression of β-actin, relative to the level at P2 (n = 3; ∗*p* ˂ 0.05 by unpaired Student’s *t* test; mean ± SEM).

To investigate the involvement of HIF1 in Schwann cell differentiation, we first examined the expression profile of HIF1α in the mouse sciatic nerve during development and after crush injury. We found that HIF1α protein is highly expressed in early postnatal development, and expression gradually becomes weaker with the progression of myelination during postnatal development (Fig. 1*A*). We also found that HIF1α expression is highly induced in the distal stump of the injured nerve after crush injury, peaking at 4 days after injury (Fig. 1*B*). Immunohistochemical co-localization of HIF1α with S100β, a Schwann cell marker, revealed that HIF1α is expressed in Schwann cell nuclei, suggesting that HIF1α-transcriptional regulation may be activated in the tissues (Fig. 1, *C* and *D*). The HIF1α protein expression profile does not resemble that of transcription factors characterizing myelinating Schwann cells (*e.g.*, Krox20) but rather is similar to that of Oct6 or molecules expressed in promyelinating Schwann cells.

**Figure 2:**
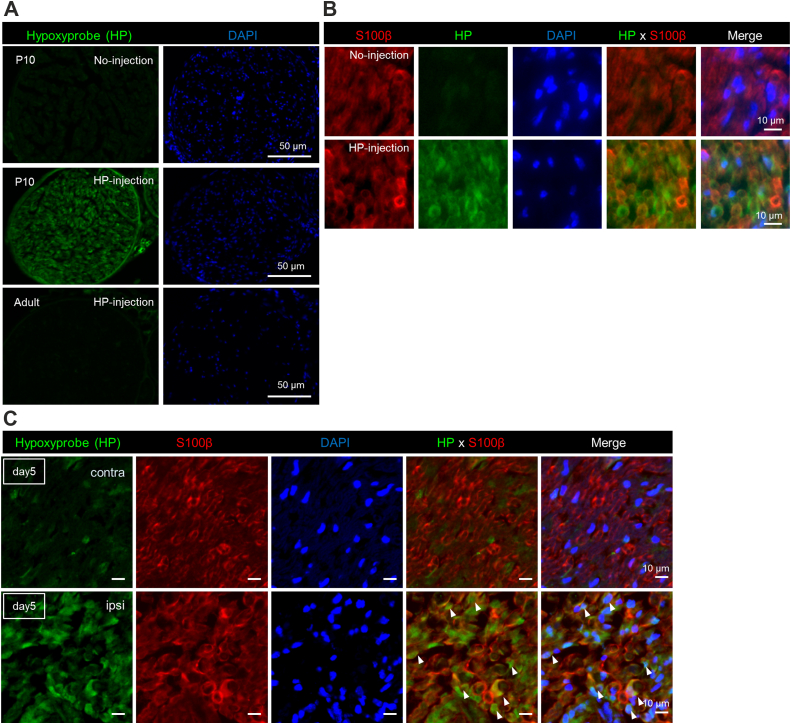
**Sciatic nerve tissue experiences hypoxia during development and after injury**. *A*, representative photomicrographs showing immunohistochemical localization of hypoxyprobe adducts in cross sections of mouse sciatic nerve tissues at P10 during development (*middle*) and in adulthood (*bottom*), shown side by side with counterstaining with DAPI. Top panels show the representative immunohistochemistry result from P10 mice without hypoxyprobe injection. (Scale bar; 50 μm). *B*, representative photomicrographs showing immunohistochemical colocalization of hypoxyprobe adducts with S100β in cross sections of mouse sciatic nerve tissues at P10 during development at high magnification view, shown side by side with DAPI counterstaining. (Scale bar; 10 μm). *C*, representative photomicrographs showing immunohistochemical co-localization of hypoxyprobe adducts with S100β in cross sections of injured (ipsi) and control (contra) adult mouse sciatic nerve tissues, shown side-by-side with DAPI counterstaining. *Arrowheads* indicate S100β-expressing Schwann cells, positive for hypoxyprobe adducts. (Scale bar; 10 μm).

Side-by-side with the expression profile analysis of HIF1α protein, we also examined the expression profile of HIF1α mRNA during development. In contrast to the dynamic expression of HIF1α protein, we found that HIF1α mRNA expression is constant or only slightly increased in postnatal development (Fig. 1*E*). The mismatch of mRNA and protein expression patterns suggests post-translational regulation of HIF1α protein, and hypoxia-dependent destabilization of HIF1α might be involved in the regulation. To examine whether PNS tissues experience hypoxia during development and after injury, we performed histological analysis using pimonidazole, a hypoxic probe (Fig. 2, *A* and *B*). We found that PNS tissues at P10 during development (Fig. 2, *A* and *B*) and Day 5 after injury (Fig. 2*C*) are both reactive for pimonidazole, suggesting that hypoxic conditions may be involved in stabilizing HIF1α protein during development and after injury in Schwann cells.

### HIF1**α** stabilization in Schwann cells promotes myelination *in vitro*

**Figure 3:**
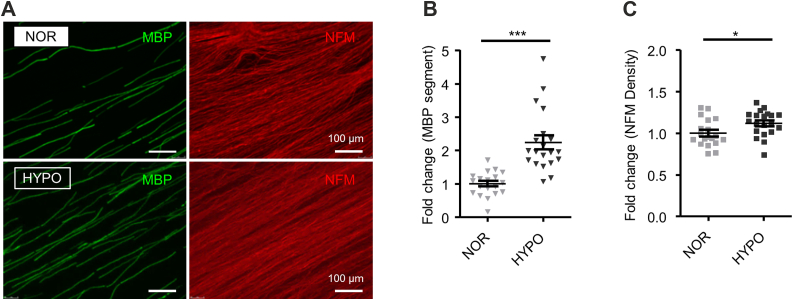
**Transient hypoxic exposure promotes myelination in mouse DRG explant culture**. *A*, mouse DRG explants were exposed to hypoxic condition (1% O_2_, 6 h). Representative photomicrographs of mouse DRG explant culture with (“Hypoxia”) or without (“Normoxia”) transient hypoxia treatment showing immunoreactivity for MBP and NFM. (Scale bars, 100 μm). *B* and *C*, quantification of the number of MBP segments (*B*) and intensity of neurofilament immunoreactivity (*C*) of mouse DRG explant culture with (“HYPO”) or without (“NOR”) transient hypoxia treatment (6 h). Images used for quantifications were captured as three or four randomly selected microscopic fields in five independent culture experiments performed for each condition. Values in *B* and *C* are shown relative to the mean value of NOR condition. (∗∗∗*p* ˂ 0.001 and ∗*p* ˂ 0.05 by unpaired Student’s *t* test; mean ± SEM).

**Figure 4:**
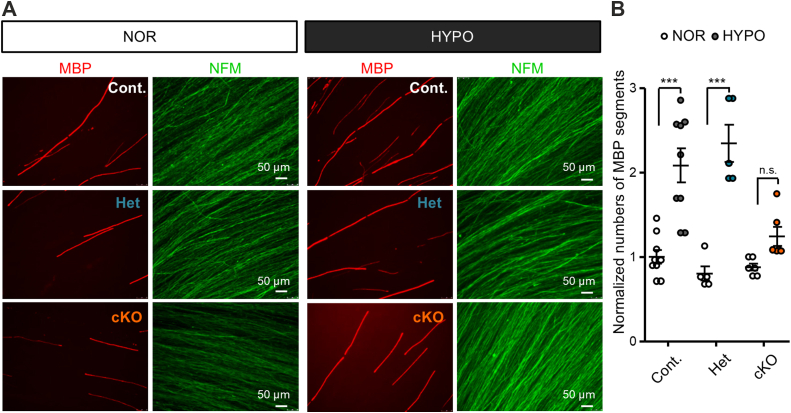
**Absence of hypoxia-induced increase in myelination in DRG explants from Schwann cell-specific HIF1α-deficient mice**. Representative photomicrographs showing immunoreactivity for MBP and NFM (*A*) and a bar graph showing quantification of the number of MBP segments in DRG explant culture generated from P0-Cre;Hif1α^Flox/Flox^ (cKO), P0-Cre;Hif1α^Flox/+^ (Het), and other littermates (Cont) with (“HYPO”) or without (“NOR”) transient hypoxia treatment (6 h). (Scale bars, 50 μm in *A*). Values in *B* are shown relative to the mean value obtained from control mice at normoxic conditions (n = 5–9; ∗∗∗*p* ˂ 0.001 by unpaired Student’s *t* test; mean ± SEM).

Nuclear localization of HIF1α in Schwann cells during development and after injury suggests that HIF1α may be functional as a transcription factor in Schwann cells. To know whether HIF1α is functional as a transcription factor in Schwann cells, we examined the effect of HIF1α induction in peripheral nerve myelination using mouse DRG explant culture. For this purpose, we first treated the myelination culture with hypoxia. With hypoxia treatment for 6 h, we found that an increased number of myelin-basic protein (MBP)-positive segments is generated (Fig. 3, *A* and *B*). To confirm that the observed hypoxia-mediated promotion of myelination is mediated by a HIF1-dependent mechanism, we utilized DRGs from conditional knock-out mice (P0-Cre;Hif1α^flox/flox^ mice) lacking HIF1α gene in P0-positive myelinating Schwann cells. We found that the hypoxia-induced promotion of myelination is not observed in the culture using DRGs from the conditional knock-out mouse (Fig. 4, *A* and *B*), suggesting that hypoxia-induced increased myelination is mediated *via* HIF1α.

To rule out the possible compensatory effect by HIF2a, another hypoxia-inducible transcription factor, we examined HIF2α expression in the nerves of HIF1α cKO mice 5 days after injury. This was done by counting the number of HIF2α-positive Schwann cell nuclei using immunohistochemistry. We found no significant difference in HIF2α expression between the cKO mice and the control (P0-Cre mice) (Fig. S1). These results suggest that a compensatory role for HIF2α in myelination in our injury model may be unlikely.

**Figure 5:**
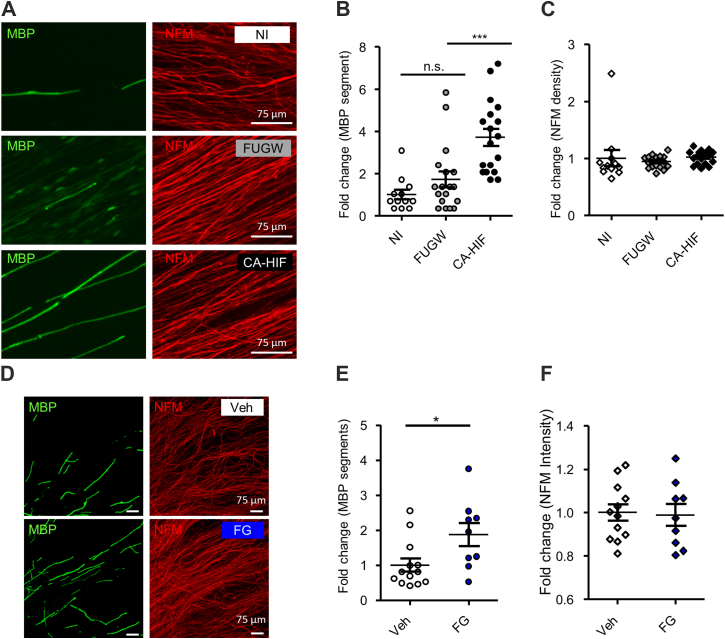
**Overexpression of stabilized HIF1α or pharmacological stabilization of HIF1α promotes myelination in mouse DRG explant culture**. *A–C*, representative photomicrographs showing immunoreactivity for MBP and NFM (*A*), quantification of the number of MBP segments (*B*) and intensity of NFM immunoreactivity (*C*) of mouse DRG explant culture with (CA-HIF1α) or without (NI: no infection; FUGW: empty vector infection) lentiviral vector-mediated overexpression of constitutively active HIF1α. (Scale bars, 75 μm). Images used for quantifications were captured as three randomly selected microscopic fields in 4 to 6 independent culture experiments performed for each condition. Values in B and C are shown relative to the mean value of NI condition. (∗∗∗*p* ˂ 0.001 by one-way ANOVA with Tukey analysis; mean ± SEM). *D–F*, representative photomicrographs showing immunoreactivity for MBP and NFM (*D*), quantification of the number of MBP segments (*E*) and intensity of NFM immunoreactivity (*F*) of mouse DRG explant culture with (FG) or without (Veh) treatment of a pharmacological HIF1α stabilizer (FG4592). (Scale bars, 75 μm) Images used for quantifications were captured as 2 to 4 randomly selected microscopic fields in 9 to 13 independent culture experiments performed for each condition. Values in *E* and *F* are shown relative to the mean value of Veh condition. (∗*p* ˂ 0.05 by unpaired Student’s *t* test; mean ± SEM).

We found hypoxia treatment also causes an increase in density of axons in explant culture, which might affect the evaluation of the effect on myelination (Fig. 3*C*). Therefore, as another means of HIF1α induction, we tried overexpression of constitutively active HIF1α (CA-HIF1α), in which two proline residues (402 and 577) were replaced by alanine (Fig. 5, *A* and *B*) or treated the myelination culture with a pharmacological stabilizer of HIF1α (FG4592 (also known as Roxadustat), an inhibitor of PHD) (Fig. 5, *D* and *E*). We found that lentivirus-mediated CA-HIF1α overexpression to the myelination culture and pharmacological stabilization of HIF1α both cause an increase in the number of MBP-positive myelin segments. In these experiments, axon density and length were unaffected (Fig. 5, *C* and *F*). As an indication of CA-HIF1α functionality, we confirmed that CA-HIF1α is located in Schwann cell nuclei (Fig. S2). We also examined the expression of glyceraldehyde-3-phosphate dehydrogenase (GAPDH), a known transcriptional target of HIF1α (10) in CA-HIF1α-overexpressing Schwann cells, and found that GAPDH expression is also upregulated by CA-HIF1α (Fig. S3). These results suggest that HIF1α may serve as a positive regulator of myelination.

### HIF1**α** regulate myelin-related gene expression

**Figure 6:**
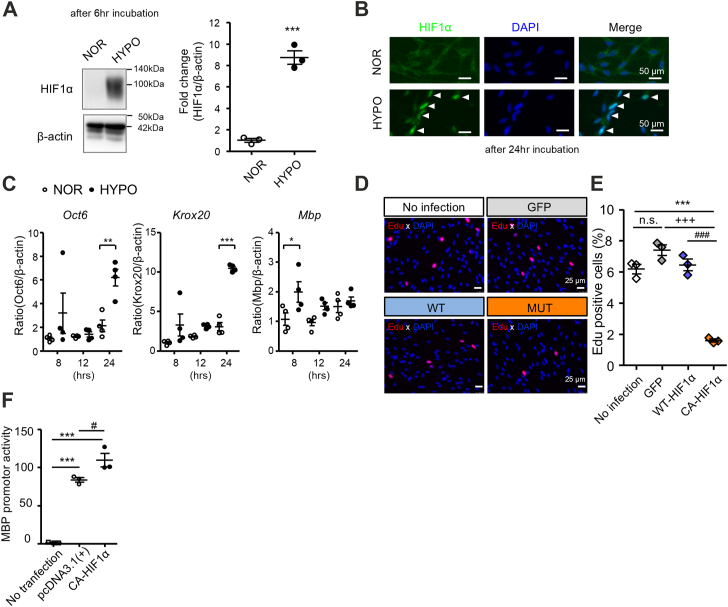
**Myelin-associated genes are transcriptionally regulated by HIF1α in Schwann cells.***A*, representative immunoblot (*left*) and a bar graph showing quantified expression levels (*right*) of HIF1α protein in primary cultured rat Schwann cells under hypoxia (HYPO) or normoxia (NOR). β-actin serves as a loading control. Quantification data are shown relative to the mean expression level at NOR condition. (n = 3; ∗∗∗*p* ˂ 0.001 by unpaired Student’s *t* test; mean ± SEM). *B*, representative photomicrographs showing HIF1α protein expression in primary cultured Schwann cells under hypoxia (HYPO) or normoxia (NOR), side-by-side with DAPI counterstained images. *Arrowheads* indicate the HIF1α immunoreactivity located in nuclei. *C*, Bar graphs showing mRNA expression levels of indicated genes in primary cultured Schwann cells maintained under hypoxia (HYPO) or normoxia (NOR) for indicated time analyzed by quantitative RT-PCR. Expression levels are normalized to β-actin, and shown relative to the levels at 8 h under NOR condition. (n = 4; ∗∗∗*p* ˂ 0.001, ∗∗*p* ˂ 0.01 and ∗*p* ˂ 0.05 by Two-way ANOVA with Bonferroni posttests; mean ± SEM). *D* and *E*, representative photomicrographs (*D*) and a bar graph showing quantified numbers (*E*) of Edu-positive primary cultured Schwann cells overexpressing GFP, wild type HIF1α (WT-HIF1α), or constitutively active HIF1α (CA-HIF1α). (Scale bar, 25 μm in *D*). The number of Edu-positive cells are shown as percentage of the total number of Hoechst-positive nuclei in *E*. (n = 4; ∗∗∗*p* ˂ 0.001, ^+++^ ˂ P0.001 and ^###^*p* ˂ 0.001 by one-way ANOVA with Tukey analysis; mean ± SEM). *F*, a bar graph for MBP promoter activity achieved by CA-HIF1α in rat Schwann cells analyzed using the dual-luciferase reporter assay system. Each column indicates the average of firefly luciferase activity downstream of the MBP promoter region divided by transgenic renilla luciferase as an internal control (n = 3; ∗∗∗*p* < 0.001 and ^#^*p* < 0.05 by one-way ANOVA with Tukey analysis; mean ± SEM). Note that in“pcDNA3.1(+)” condition, Schwann cells were transfected with the MBP- luciferase plasmid, containing MBP promoter region, together with pcDNA3.1(+) empty vector. Therefore, basal-level MBP promoter activity in cultured Schwann cells is detected in“pcDNA3.1(+)” condition, and the increase between “pcDNA3.1(+)” and “CA-HIF1a” corresponds to the transcriptional activity of HIF1α.

To gain mechanistic insights on HIF1α-mediated promotion of myelination, we tried to determine the genes transcriptionally regulated by HIF1α. To this end, we first examined regulation of myelin-related genes in Schwann cells when HIF1α overexpression is induced. To induce HIF1α expression in Schwann cells, primary Schwann cells were cultured under hypoxic conditions. HIF1α protein was detected in Schwann cells exposed to hypoxia for 6 h (Fig. 6*A*), and stable expression of HIF1α in the Schwann cell nucleus was observed after 24 h of hypoxia exposure (Fig. 6*B*). We found that hypoxia significantly up-regulates the expression of Oct6, Krox20 and Mbp (Fig. 6*C*). To assess the potential role of HIF1α in Schwann cell proliferation, we determined cell proliferation using the EdU incorporation assay (Fig. 6, *D* and *E*). We found that the number of Edu-positive nuclei is decreased by overexpression of CA-HIF1α compared to those of EGFP or wild type HIF1α, suggesting that HIF1α stabilization may repress Schwann cell proliferation.

**Figure 7:**
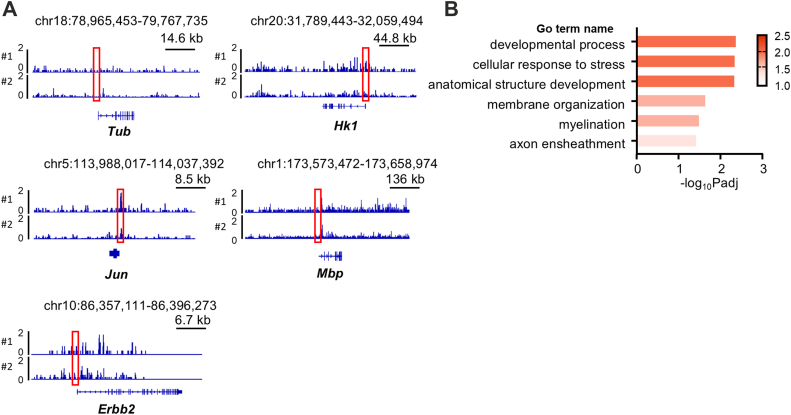
**ChIL-seq detects genes transcriptionally regulated by HIF1α in Schwann cells**. *A*, the Integrative Genomics Viewer (http://www.broadinstitute.org/igv/) -assisted visualization of ChIL–seq signals are shown for the indicated genes in duplicate. Primary rat Schwann cells were exposed to hypoxia for 18 h and subjected to ChILs-seq analysis. Tub and Hk1 were also shown as a negative- and a positive-control, respectively. Note the accumulation of HIF1α association sites in proximity of jun, mbp, and erbb2 genes (boxed in *red*). *B*, pathway analysis of HIF1α-regulated Schwann cell-specific genes by g:Profiler (https://biit.cs.ut.ee/gprofiler/gost). Hk1, hexokinase 1; Mbp, myelin basic protein; Tub, tubulin.

**Figure 8:**
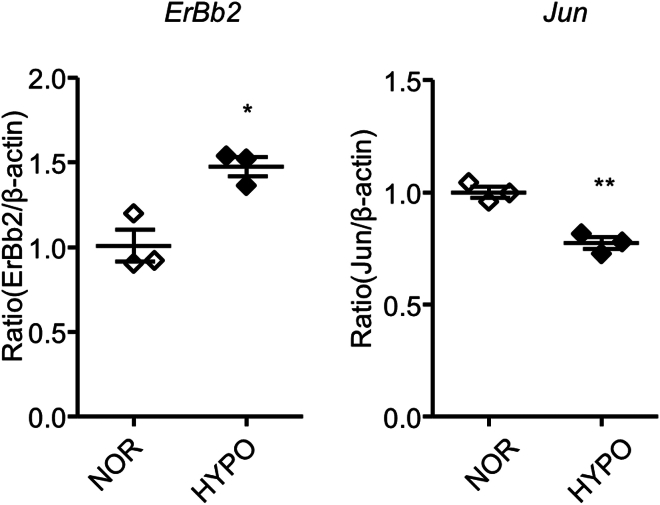
**Expression of myelin-related genes identified by ChIL-seq analysis is regulated by hypoxia in cultured Schwann cells**. Quantified expression levels of Erbb2 and c-Jun in primary cultured rat Schwann cells treated with hypoxia for 12 h (HYPO) or normoxia (NOR) analyzed by quantitative RT-PCR. The expression levels were normalized to that of β-actin, and shown relative to the mean levels in NOR condition. (n = 3; ∗∗*p* ˂ 0.01 and ∗*p* ˂ 0.05 by unpaired Student’s *t* test; mean ± SEM).

For unbiased screening of HIF1α target genes in Schwann cells, we performed a comprehensive analysis using Chromatin integration labeling followed by sequencing (ChIL-seq). To support the specificity of ChIL-seq signals, we conducted GO-based stratification of genes according to the presence or absence of a hypoxia response element (HRE) motif within ±2 kb of the transcription start site (TSS). Among HRE-positive genes, ChIL-positivity exceeded 97% across all tested GO categories, including myelination, membrane organization, and development. These results suggest that the observed peaks reflect *bona fide* promoter-binding events mediated by HRE-dependent recruitment (Fig. 7 and Table 1). Among the myelination-related genes, we found myelin basic protein (Mbp), epidermal growth factor receptor tyrosine kinase 2 (ErbB2), and c-jun were all identified in ChIL-seq. Hexokinase-1, a well-known HIF1α-regulated gene was also detected. These results were confirmed in hypoxia-exposed Schwann cells with qPCR analyses (Fig. 8). To verify that these identified genes are directly regulated by HIF1α, we performed a luciferase assay using rat primary Schwann cells. We found that overexpression of CA-HIF1α could enhance Mbp transcription (Fig. 6*F*). These results suggest that HIF1α may induce myelin-related gene expression and thereby promote Schwann cell differentiation.

**Table 1:** GO-based stratification of ChIL-positivity rates among genes containing hypoxia response elements (HREs)

GO term	ChIL-positive & HRE + genes	HRE + genes	ChIL-positivity rate	Total GO genes
Myelination	40	41	98%	175
Membrane organization	346	353	98%	1879
Developmental process	59	60	98%	289
Cellular response to stress	352	358	98%	1471
Axon ensheathment	2	2	100%	9
Anatomical structure development	31	31	100%	99

ChIL-positivity rates among HRE-containing genes were calculated for each gene category associated with the indicated GO terms.

### Schwann cell HIF1**α** promotes re-myelination and sensorimotor recovery after nerve injury

**Figure 9:**
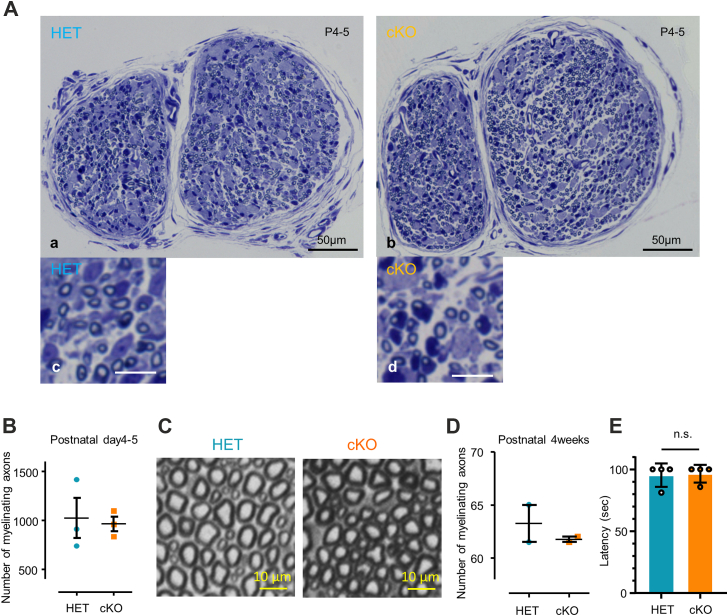
**Peripheral nerves of P0-Cre;Hif1αFlox/Flox mice develop normally**. *A*, representative photomicrographs of sciatic nerve semithin cross-sections from P0-Cre: HIF1α^flox/flox^(cKO; b,d) and P0-Cre: HIF1α^flox/+^ (HET; a,c) mice at P4-5, with low (a,b) and high (c,d) magnification views. (Scale bars, 50 μm in a and b; 10 μm in c and d). *B*, quantification of total number of myelinated axons in sciatic nerve semithin cross-sections exemplified in (*A*) from P0-Cre: HIF1α^flox/flox^(cKO) and P0-Cre: HIF1α^flox/+^ (HET) mice at P4-5. n = 3. *C*, representative photomicrographs of sciatic nerve semithin cross-sections from P0-Cre: HIF1αflox/flox(cKO) and P0-Cre: HIF1αflox/+ (HET) mice at 4 weeks of age. (Scale bars, 10 μm). *D*, quantification of the number of myelinated axons within a randomly selected 50 μm × 50 μm area in each section on the sciatic nerve semithin cross-sections exemplified in (*C*) from P0-Cre: HIF1α^flox/flox^(cKO) and P0-Cre: HIF1α^flox/+^ (HET) mice at 4 weeks of age. Each plot represents the average number of myelinated axons in two randomly selected areas. (n = 2). *E*, a bar graph showing wire hang test results from P0-Cre: HIF1α^flox/flox^(cKO) and P0-Cre: HIF1α^flox/+^ (HET) mice at 4 weeks of age. (n = 4 for each genotype).

**Figure 10:**
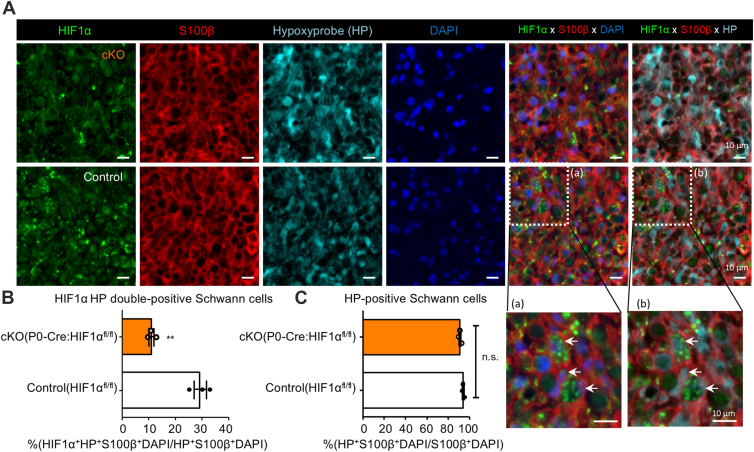
**Reduced number of HIF1α HP double-positive Schwann cells in injured nerve demonstrates efficient conditional deletion of HIF1α gene in P0-Cre: HIF1α^flox/flox^ mice.** Representative photomicrographs (*A*) and quantified number (*B* and *C*) of Schwann cells showing hypoxia-induced HIF1α activation in adult P0-Cre: HIF1α^flox/flox^ (cKO) mice or HIF1α^flox/flox^ (Control) mice 5 days after crush injury. Significantly reduced number of HIF1α HP double-positive Schwann cell nuclei (exemplified as a and b in *A*) in cKO mice despite the equivalent percentage of HP-positive Schwann cells demonstrates the efficient, conditional deletion of HIF1α gene in Schwann cells. (Scale bar, 10 μm in *A*. n = 3; ∗∗*p* ˂ 0.01 by unpaired Student’s *t* test; mean ± SEM in *B* and *C*).

**Figure 11:**
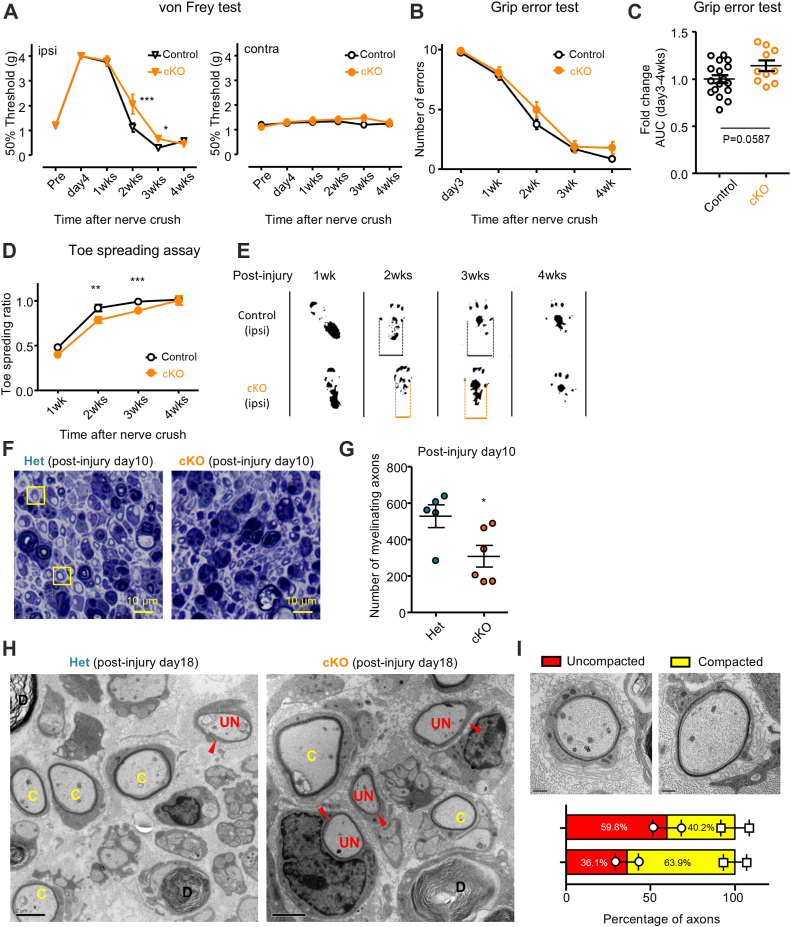
**Mice lacking Schwann cell HIF1α showed slow sensorimotor recovery after nerve injury**. Behavioral analyses of P0-Cre;Hif1αFlox/Flox (cKO) and Hif1αFlox/Flox littermates (Control) after sciatic nerve crush injury by von Frey test (*A*), grip error test (*B* and *C*), and toe spreading assay (*D* and *E*). Each test was performed at the indicated time point in (*A*, *B*, and *D*). Von Frey test data were obtained from hindpaws on both injured (ipsi) and uninjured (*contra*) sides in (*A*). Grip error test data were analyzed as values of the area under the curve (AUC) shown in (*B*) relative to the mean AUC of the control mice shown in *C*. Representative footprint images obtained for toe spreading assay at 1 to 4 weeks after injury are shown in *E*. (n = 6–14, ∗∗∗*p* ˂ 0.001, ∗∗*p* ˂ 0.01 by Two-way ANOVA with Bonferroni posttests; mean ± SEM). *F* and *G*, rrepresentative photomicrographs of sciatic nerve semithin cross-sections from adult P0-Cre;Hif1αFlox/Flox (cKO) and P0-Cre;Hif1αFlox/+ (Het) mice 10 days after nerve crush stained with toluidine *blue* (*F*), and quantification of the number of all the myelinated axons of each genotype (*G*). (Scale bar, 10 μm in *F*) (n = 4 for heterozygous and six for cKO, ∗*p* ˂ 0.05 by unpaired Student’s *t* test; mean ± SEM). *H* and *I*, representative electron micrograph of sciatic nerve cross-section from adult P0-Cre;Hif1αFlox/Flox (cKO) and P0-Cre;Hif1αFlox/+ (Het) mice 10 days after nerve crush. “*C*” indicates axons wrapped by tightly compacted myelin sheath. “UN” indicates axons without compacted myelin sheath (uncompacted). (Scale bar, 2 μm in *H*) High power view of representative axons with compacted and uncompacted myelin sheath are shown in *I*. (Scale bar, 0.5 μm) The percentage of axons with compacted and uncompacted myelin sheath analyzed from 150 to 200 axons on electron micrographs obtained from two mice of each genotype are also shown.

Our results suggested that HIF1α positively regulates peripheral nerve myelination. To further explore HIF1α function in myelin formation, we employed P0-Cre;Hif1α^flox/flox^ mice and analyzed development and degeneration/regeneration of peripheral nerves. P0-Cre;Hif1α^flox/flox^ mice exhibited no apparent defects in the myelin morphology of peripheral nerves during development and in adulthood (Fig. 9, *A*–*D*). We also found that P0-Cre;Hif1α^flox/flox^ mice did not show motor defects in the wire hang motor behavior test (Fig. 9*E*). These observations suggest that Schwann cell HIF1α may be not required for the formation and maintenance of myelin in peripheral nerves. To examine the possibility that loss of Hif1α in adult Schwann cells affects remyelination of peripheral nerves, we analyzed the time course of sciatic nerve degeneration/regeneration after crush injury. HIF1α expression was markedly reduced in injured nerves of P0-Cre;Hif1α^flox/flox^ mice compared with those of Hif1α^flox/flox^ littermates (Fig. 10). To confirm Schwann cell-specific HIF1α deletion, we performed immunohistochemical analysis of sciatic nerve tissue using antibodies against CD34 (a marker for vascular endothelial cells and the perineurium), S100β (a Schwann cell marker) and HIF1α and quantified the proportion of HIF1α-positive cells in endothelial cells (defined as HIF1α+CD34+DAPI+/CD34+DAPI+) and Schwann cells (HIF1α+S100β+DAPI+/S100β+DAPI+). We found that the number of HIF1α-positive Schwann cells is markedly reduced in cKO mice compared to controls, while HIF1α-positive vascular endothelial cell proportion in cKO is comparable to that in control, indicating the Schwann cell-specificity of HIF1α deletion (Fig. S4). We examined sensorimotor function after nerve injury in P0-Cre;Hif1α^flox/flox^ mice using behavioral tests including von Frey test, grip test, and toe spreading assay (Fig. 11). We found that P0-Cre;Hif1α^flox/flox^ mice show delayed recovery from injury-induced sensory impairment at 2 to 3 weeks post injury time points but catch up by 4 weeks (Fig. 11, *A*–*E*). To analyze the impaired recovery at a cellular level, we examined microscopic morphology of the P0-Cre;Hif1α^flox/flox^ mouse injured nerves. We found that the number of myelinating axons in regenerating nerve is significantly fewer in P0-Cre;Hif1α^flox/flox^ mice than those in heterozygous mice (Fig. 11, *F* and *G*). We also found by electron microscopic analysis that the frequency of axons with uncompacted myelin (incomplete myelin or only one layer of myelin) is higher in P0-Cre;Hif1α^flox/flox^ mice than heterozygous mice (Fig. 11, *H* and *I*). These observations suggest that loss of Schwann cell HIF1α results in a delayed recovery from sensorimotor impairment and re-myelination after injury.

### HIF1**α** stabilizer ameliorates sensorimotor dysfunction after nerve injury

**Figure 12:**
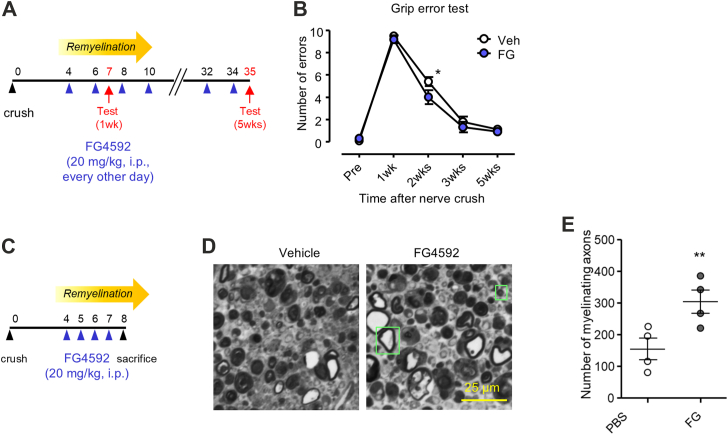
**HIF1α stabilizer accelerates recovery from peripheral nerve injury**. *A* and *B*, grip error tests data (*B*) from adult mice at indicated time points after sciatic nerve crush injury treated with a pharmacological HIF1α stabilizer (FG4592; FG) or vehicle (Veh) every other day following the schedule shown in *A*. (n = 10, ∗*p* ˂ 0.05 by unpaired Student’s *t* test; mean ± SEM). *C–E*, representative photomicrographs (*D*) of sciatic nerve cross-semithin sections of adult mice 8 days after crush injury treated with FG4592 (FG) or vehicle (Veh) every day following the schedule shown in *C*. Quantified numbers of all the myelinating axon of four mice for each treatment group are shown in *E*. (∗∗*p* ˂ 0.005 by unpaired Student’s *t* test; mean ± SEM).

The myelination-promoting effect of HIF1α in nerve regeneration *in vivo* suggests that HIF1α stabilization may promote nerve regeneration. To examine this possibility, we administered a pharmacological HIF1α stabilizer FG4592 on the mouse sciatic nerve crush injury model and analyzed the time course of nerve regeneration. We found that FG4592 treatment significantly promotes sensorimotor recovery as demonstrated by improved performance in grip test results (Fig. 12, *A* and *B*). Histologically, we found increased number of myelinated axons by FG4592 treatment (Fig. 12, *C*–*E*). These results suggest that HIF1α stabilization could have a therapeutic effect in the recovery of sensorimotor impairment in PNS by promoting remyelination.

## Discussion

In this study, we showed that Schwann cell HIF1α protein might enhance peripheral myelination after nerve injury. Our data suggest that HIF1α facilitates Schwann cell differentiation as a transcription factor. We found that hypoxia not only promotes Schwann cell differentiation but also reduces proliferation. Based on the comprehensive analysis of HIF1α target genes, we found that expression of myelin-related genes such as *Erbb2* and *c-jun* in Schwann cells by hypoxic exposure. HIF1α stabilization increased *Erbb2* expression, and decreased *c-jun* expression in Schwann cells. Schwann cell myelination depends on promyelin factors including ErbB2 (16); while c-Jun represents a signal that functionally stands in opposition to the pro-myelin factors, negatively regulating the myelinating Schwann cell phenotype (17). Schwann cell HIF1α in response to hypoxia might simultaneously regulate both the accelerator and the brake of gene expression that controls myelination. On the other hand, microscopic and immunochemical analysis of sciatic nerves of P0-Cre;Hif1α^flox/flox^ mice after injury revealed a delay in Schwann cell differentiation and remyelination. The POU transcription factor Oct6 has been implicated as a major transcriptional regulator in Schwann cell differentiation (4, 5). Oct6-deficient Schwann cells have been known to exhibit a delay in Schwann cell differentiation and myelination with a transient arrest at the pro-myelination stage (18). Thus, HIF1α appears to be required for the transition of pro-myelin cells to myelinating cells. Further studies are needed to determine roles for HIF1α as a regulator of Schwann cell differentiation.

It is well known that transcriptional activation of erythropoietin is mediated by HIF1α (19). Sanjay *et al.* showed that axon injury stimulates the production of erythropoietin (EPO) in Schwann cells, which inhibits axonal degradation by activating neuronal erythropoietin receptors (EPORs) (20). Campana *et al.* demonstrated that the EPO signaling reduces TNFα mRNA expression in Schwann cells and improves neuropathic pain symptoms (21). These findings suggest that HIF1α-dependent pathways could be therapeutic targets for peripheral neuropathy. Our finding proposes that HIF1α represents a critical regulator of myelination and suggests hypoxia as a tool to stimulate peripheral nerve myelination.

## Experimental procedures

### Animals and surgery

All experimental procedures using mice and rats were approved by the Animal Welfare Committee in the National Center of Neurology and Psychiatry. The animals were housed under controlled ambient temperature (23–24 °C, 60–70% humidity) with a 12-h dark/light cycle and given water and food ad libitum. All mice were on a C57BL/6 background. Mice and rats were purchased from CLEA Japan.

To generate Schwann cell-specific HIF1α knockout mice, HIF1α^flox/flox^ mice were crossed with P0-Cre transgenic mice to obtain P0-Cre; HIF1α^flox/+^ (hetero; heterozygous) males and females (22, 23). Each heterozygote was further crossed to obtain P0-Cre; HIF1α^flox/flox^ (cKO; conditional knockout) mice. To generate a peripheral nerve injury model, the sciatic nerve of male mice (8–10 weeks) was crushed as previously described (24, 25).

### *In vitro* myelination assay using DRG explant culture

*In vitro* myelination cultures were generated using mouse DRG explants as previously described (26). Briefly, DRGs were dissected from E12 embryos of C57BL/6J mice (CLEA Japan), plated onto 24 well plates coated with poly-L lysine (100 μg/ml, Sigma Aldrich) and laminin (Cultrex mouse laminin I, 2.5 μg/ml, R&D systems, #: 3400-010-01), and cultured in MACS Neuro Medium (Miltenyi Biotec, 130-093-570) supplemented with 10% fetal bovine serum (FBS) (Bio sera), 1:200 Glutamax (Thermo Fischer Scientific, 35050061), 50 U/ml Penicillin/Streptomycin (Thermo Fischer Scientific, 15070063), 100 ng/ml 2.5S nerve growth factor (NGF) (COSMO BIO, CLMCNET-005.1) and 0.1 mM sodium pyruvate solution (Wako, 190-14881). Sixteen hours later, the medium was changed to MACS Neuro Medium supplemented with 1% MACS NeuroBrew-B21 (Miltenyi Biotec, 130-093-566), 1:200 Glutamax, 50 U/ml Penicillin/Streptomycin and 100 ng/ml 2.5S NGF. On day 5, the medium was changed into DMEM/F12 (Wako, 048-29785) supplemented with 1:100 N2 supplement (R&D systems, AR009), 50 U/ml Penicillin/Streptomycin and 100 ng/ml 2.5S NGF and maintained for 3 to 5 days. On day 8 to 10, the medium was replaced with DMEM (Sigma, D5030) supplemented with 10% FBS, 1:200 Glutamax, 50U/ml Penicillin/Streptomycin and 100 ng/ml 2.5S NGF, and 50 μg/ml of L-ascorbic acid (Sigma Aldrich) to induce myelination.

### Schwann cells culture

Primary Schwann cells were prepared from the sciatic nerves of neonatal rats (2 days old) according to previous reports (25, 27). Briefly, the dissected sciatic nerves were digested with collagenase (Worthington Biomedical, CLS-1) and dispase (Collaborative, 40235) for dissociation. Schwann cells were purified by treatment with 10 μM cytosine arabinoside (Wako), followed by complement-mediated cytolysis using anti-Thy1.1 (Serotec) and rabbit complement (Cappel Laboratories) to remove fibroblasts. Schwann cells were cultured with DMEM (Wako, 044-29765) supplemented with 10% FBS, 2 μM forskolin (Sigma Aldrich, F6886), and 10 ng/ml Hereglin β1 (EGF domain) (Sigma, H7660), and used for experiments after three or more passages. For experimental data using primary cultures of rat Schwann cells, a sample collected from one dish or one well per condition was regarded as a single sample.

### Cell culture in a hypoxic environment

For hypoxic treatment, cells were cultured in a hypoxic chamber (CO2 Incubator 9000EX, WAKEN B TECH) or in a humidified chamber with AneroPack (MITSUBISHI GAS CHEMICAL) according to the manufacturer’s instructions. The oxygen concentration was set at 1% using an oxygen indicator. Hypoxic incubators were employed in the experiments shown in Figures 3 and 4, and AneroPacks were used in those presented in Figures 6 and S3. Note that the use of the AnaeroPack system to generate a hypoxic experimental environment might lead to increased CO_2_ levels in the atmosphere, which could influence the degree of HIF1α stabilization directly and/or *via* changes in the pH of the culture medium. For the experiments using AneroPacks, HEPES was added to the culture medium at a final concentration of 25 mM to mitigate potential pH fluctuations.

### Plasmid construction

The full-length mouse HIF1α cDNA was amplified by PCR from corresponding cDNA clones (Horizon Discovery Ltd, Clone ID: 4019056) and inserted into a pcDNA3.1(+) expression vector. To generate the expression construct for constitutively active HIF1α protein, two prolines (P402 and P577) were mutated to alanine by PCR-mediated site-directed mutagenesis using Pfu enzyme (Agilent) (28). Generation of MBP-firefly luciferase expressing pxP1 for luciferase assay was previously described. T7 and SV40-renilla luciferase expressing psiCHECK-2 vectors were purchased from Promega (C8021). The integrity of the plasmids was confirmed by sequence analysis.

### Gene transfection by electroporation and infection using adenovirus or lentivirus

For transfection into primary Schwann cells, electroporation using the NEPA21 electroporator (NEPA GENE) was performed according to the manufacturer’s instructions. For adenovirus-mediated gene transfer, the pAxCAwtit2 cosmid vector was used for expression vector construction. Adenovirus was generated using the Takara adenovirus expression kit (Takara Bio) according to the manufacturer’s instructions. The vector solution was concentrated by Vivaspin (Sartorius) according to the manufacturer’s instructions. For lentiviral infection, constitutively active HIF1α (P402A and P577A) or wild type HIF1α were cloned into the FUGW vector (29). Lentiviral packaging was performed using HEK293T cells as described in our previous report (30).

### Cell proliferation assay

For the cell proliferation assay, Edu (final concentration 50 μM) was added to the cell culture 2 h before PFA fixation for detection by Clik-iT EdU labeling Kit (Invitrogen) according to the manufacturer’s instructions. For quantification of proliferating cells, the number of Edu-labeled cells was counted under a microscope in five randomly selected fields per well for each condition and normalized by the total number of cells evaluated by counterstaining with Hoechst dye.

### Luciferase reporter assay in Schwann cells

The luciferase-expressing plasmids were co-transfected with constitutively active HIF1α (P402A and P577A) inserted into the pcDNA3.1(+) or control pcDNA3.1(+) vector by electroporation. After the Schwann cells were cultured in a 60 mm dish for 3 days, luciferase activity was determined using the Dual-Luciferase Reporter Assay System (Promega, E1910). Each sample from one plasmid electroporation was regarded as a single experiment, and three independent experiments were performed.

### Immunoblot

Tissue or cell samples were homogenized in SDS-containing sample buffer (2% SDS, 10% Glycerol, 50 mM Tris-HCl, PH = 6.8) with protease inhibitor and phosphatase inhibitor (Nacalai Tesque). For experiments using sciatic nerves, tissue lysate obtained from 6 to 7 pieces of 2 day-old, five pieces of 10 day old, and two pieces of 21 days or older mice were used as a single sample. Lysate containing 30 μg proteins per sample was separated by SDS-PAGE and transferred onto a PVDF membrane. The membrane was blocked with Blocking One (Nacalai Tesque, 03953-95) at room temperature for 30 min and incubated with a primary antibody diluted in the blocking buffer at 4 °C overnight. The membrane was then washed with Tris-buffered saline containing 0.1% Tween-20 and incubated with a secondary antibody diluted in blocking buffer at room temperature for 2 h. Immunoreactivity was detected using a chemiluminator with an enhanced chemiluminescent substrate for the detection of horseradish peroxidase (FUJIFILM, ImmunoStar). The image was analyzed using ImageJ software (National Institutes of Health).

### Quantitative reverse transcription (RT)-PCR

Total RNA was purified from samples using TRI Reagent (Molecular Research Center) and converted to cDNA using ReverTra Ace (TOYOBO) using random primers. The resultant cDNA was used as a template for quantitative real-time PCR with the THUNDERBIRD qPCR Mix (TOYOBO) using the Applied Biosystems Prism model 7300 sequence detection instrument and a standard SYBR green detection protocol. The definition of the number of samples is the same as the one for immunoblot. The following primers were used for PCR amplification:

β-actin forward 5′-AGGCCATGTACGTAGCCATCCA-3′, reverse 5′-TCTCCGGAGTCCATCACAATG-3′

Oct6 forward 5′- TACCGCGAAGTGCAGAAGC-3′, reverse 5′- CGTGGGTAGCCATTGAGGG-3′

Krox20 forward 5′-TCTCCGTGCCAGAGAGATCC-3′, reverse 5′-GAGGGCAGGGGAACGGCTTT-3′

Mbp forward 5′-ACTCACACACAAGAACTACCCA-3′, reverse 5′-AGCTAAATCTGCTGAGGGACA-3′

c-jun forward 5′-CGGGCTGTTCATCTGTTTGT-3′, reverse 5′-CCGGGACTTGTGAGCTTCTT-3′

Erbb2 forward 5′-TGACAAGCGCTGTCTGCCG-3′, reverse 5′-CTTGTAGTGGGCGCAGGCTG-3′

### Chromatin integration labeling followed by sequencing (ChIL-seq)

ChIL analysis was performed as previously reported (31). Briefly, rat Schwann cells were exposed to hypoxia and were immunostained using an anti-HIF1α antibody (Enzo, ALX-804-216). After labeling with an oligonucleotide-conjugated secondary antibody (ChIL probe), the nearby genome sequence is amplified by Tn5 transposase-mediated transposition followed by T7 RNA polymerase-mediated transcription. The synthesized RNA was purified using the RNeasy MinElute Cleanup Kit (Qiagen, 74204), and cDNA was synthesized from purified RNA using a Verso cDNA Synthesis Kit (Thermo Fisher, AB1453A). After sequencing, the reads were cleaned by Trim Galore (version 0.6.10) and mapped to rn6 with Bowtie2 (version 2.3.1).

Bigwig files for visualization were generated by deepTools (version 3.5.4) with the following command “bamCoverage --binSize 100 –normalize Using CPM”.

### Morphological analysis

For immunohistochemistry using sciatic nerves (32), tissue sections were cut on a cryostat and subjected to dehydration fixation with ice-cold methanol. For immunocytochemistry using cultured Schwann cells, cells were fixed with 4% paraformaldehyde (Wako) in PBS for 10 min, and permeabilized with ice-cold methanol at −30 °C for 20 min. For hypoxyprobe staining, mice were transcardially perfused with 4% paraformaldehyde/PBS for fixation, and the sciatic nerve was isolated. Sections/cells were incubated with anti-pimonidazole antibody (Hypoxyprobe Inc.) for overnight incubation at 4 °C, followed by secondary antibody for 2 h at room temperature. Antibodies were diluted in Blocking One. Sections/cells were counterstained with 4,6-diamidino-2-phenylindole.

For evaluation of myelination in culture, the number of MBP-positive segments was counted in 3 to 6 randomly selected microscopic fields per each sample, and the indicated numbers of samples were used for each condition.

Procedures for toluidine blue staining and electron microscopy analysis of sciatic nerve tissues were previously described (33). Briefly, mice were transcardially perfused with 4% paraformaldehyde/PBS, and sciatic nerve tissues were dissected and post-fixed with 1.4% paraformaldehyde (Wako) and 1% glutaraldehyde in PBS overnight. For toluidine blue staining, tissues were dehydrated and embedded in plastic for cross sections on an ultramicrotome. For electron microscopy analysis, tissues were treated with 1% Osmium (VIII) Oxide Solution (FUJIFILM) in PBS for 30 min. The samples were stained with 3% uranyl acetate for 60 min, serially dehydrated, and embedded in epon812 (TAAB). Ultrathin sections cut to 70 nm were observed by transmission EM (Tecnai Sprit, FEI/Thermo Fisher Scientific).

## Antibodies

The antibodies used in each experiment were as follows: anti-HIF1α antibodies (for Western blotting—rabbit polyclonal antibody from Gene Tex, GTX127309, RRID: AB_2616089, rabbit monoclonal antibody from Cell Signaling Technology, 36,169; for immunocytochemistry/immunohistochemistry—rabbit monoclonal antibody from Abcam, ab179483; for ChIL—mouse monoclonal antibody from Enzo, ALX-804-216); anti-HIF2α antibody (for immunohistochemistry-rabbit polyclonal antibody from Abcam, ab109616); anti-Myelin basic protein (for immunocytochemistry—rat monoclonal antibody from Merck, MAB386); anti-Neurofilament M (for immunocytochemistry—rabbit polyclonal antibody, AB1987); anti-S100β (for immunohistochemistry—mouse monoclonal antibody from Sigma, S2532); anti-pimonidazole antibody (for immunohistochemistry—rabbit antibody from Hypoxyprobe Inc, PAb2627AP); anti-β-actin (for Western blotting—mouse monoclonal antibody from Sigma, A5441 and rabbit polyclonal antibody from BioLegend, 622101, RRID: AB_315945). Horseradish peroxidase (HRP)-conjugated secondary antibodies (Jackson Immunoresearch), and Alexa fluor-conjugated antibodies (Thermo Scientific) were used for detection. For all experiments involving antibody reactions, we confirmed that no immunoreactive signal was observed without primary antibodies.

### Behavioral tests

The von Frey test was performed by applying calibrated von Frey filaments (bending forces of 0.04, 0.07, 0.16, 0.4, 0.6, 1, 2, and 4 g) to the middle of the plantar surface of the hind paw. Paw withdrawal response was recorded, and 50% threshold was calculated using the Up-Down Reader software and associated protocol (34).

Toe spread assay and grip assay were performed as previously described (33). In the toe spread assay, mouse foot pads were painted with black pigment ink. The mice were made to walk through a narrow tunnel laid over a blank paper. The distance between the first and fifth digits was measured. In the grip assay (35), mice were placed on the wire mesh, and the number of errors (the number of times they stepped off during 10 steps) was recorded. All behavioral experiments were conducted in a blind fashion.

### Drug administration

FG4592 (Cayman Chemical, 808118-40-3) was intraperitoneally injected starting from 4 days after sciatic nerve injury after nerve injury, every other day (for motor function analysis) or every day (for morphological analysis of remyelinated axons) at 20 mg/kg. The administration dosage was determined by following previous reports (36).

Pimonidazole HCl was injected intraperitoneally at 60 mg/kg (37). In the nerve injury model, pimonidazole HCl was injected 5 days after injury. The mice were sacrificed for analysis 4 h after the injection.

### Statistical analysis

Statistical analyses were performed using GraphPad Prism (GraphPad Software, Inc). We used all the data obtained from each experiment and performed randomization methods in all experiments. Data are presented as mean ± S.E. in all graphs. The results were analyzed using One-way analysis of variance, followed by *post hoc* Tukey’s test. Time course data for behavioral experiments were analyzed by Two-way analysis of variance. After confirming statistical significance, Bonferroni multiple comparison tests were performed. The results of cell measurements were analyzed using an unpaired Student’s *t* test (two-tailed). A threshold for statistical significance was set at *p* < 0.05.

## Data availability

All data analyzed during this study are included in this paper and its supplementary information file. ChIL-seq data in this study have been deposited in GEO, maintained by the NCBI under accession number GSE254040. All other data are available on request by contacting the corresponding author (taraki@ncnp.go.jp).

## Supporting information

This article contains supporting information.

## Conflict of interest

The authors declare that they have no conflicts of interest with the contents of this article.
