# Supplementary material for: Hypoxia-inducible factor 1α in Schwann cells promotes peripheral nerve myelination
Source: J Biol Chem. 2025 Jul 1;301(8):110433. doi: 10.1016/j.jbc.2025.110433 (PMC12309608; doi:10.1016/j.jbc.2025.110433)
Supplement: Supplemental Figures [file mmc1.pdf]

Supplementary Figure 1

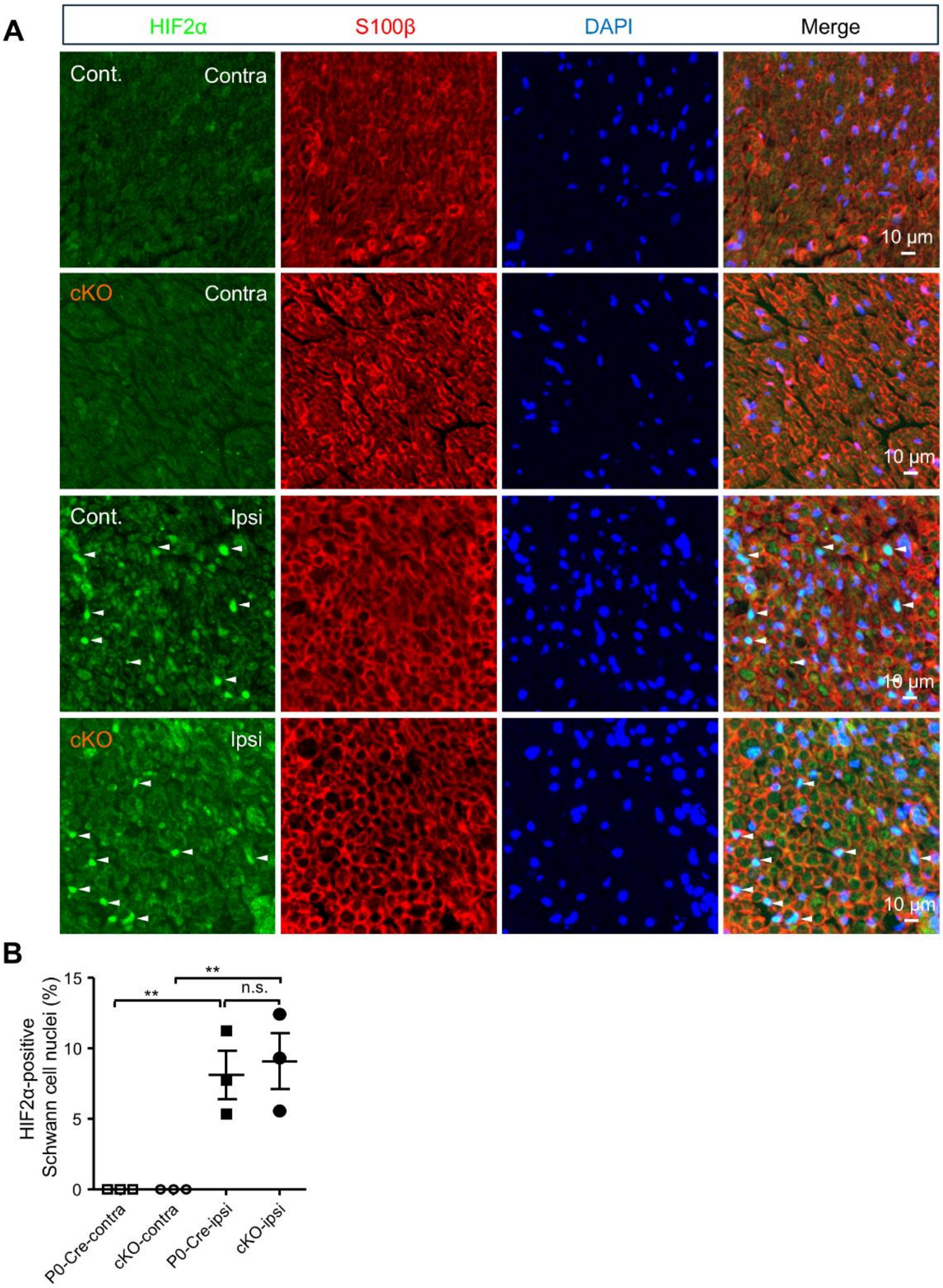

A) Representative photomicrographs showing immunohistochemical expression of HIF2α in cross sections of post-injury day5 (Ipsi) and intact (Contra) sciatic nerve from adult HIF1α cKO mice, shown side-by-side with S100β and DAPI counterstaining. Arrowheads indicate S100β-expressing Schwann cells, positive for hypoxyprobe adducts. (Scale bar; 10 μm). B) The percentage of HIF2α positive

Schwann cells nuclei in total Schwann cells nuclei. (n=3; **P <0.01 one-way ANOVA with Tukey analysis; mean ± SEM).

Supplementary Figure 2

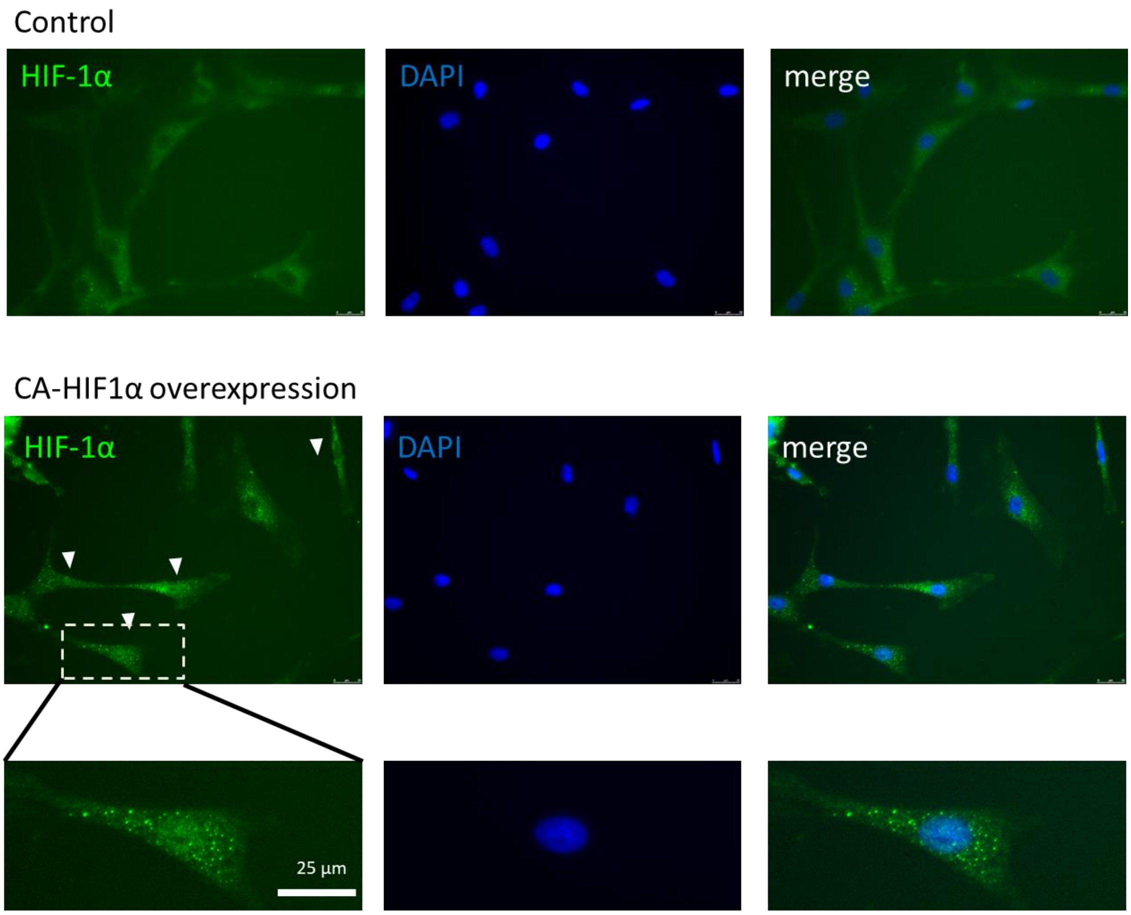

Overexpression of CA- HIF1αincreases nuclear HIF1α.

Schwann cells were overexpressed with CA-HIF1α, and subject to immunocytochemistry to visualize HIF1α. Note that overexpressed CA-HIF1α is localized to nucleus.

Supplementary Figure 3

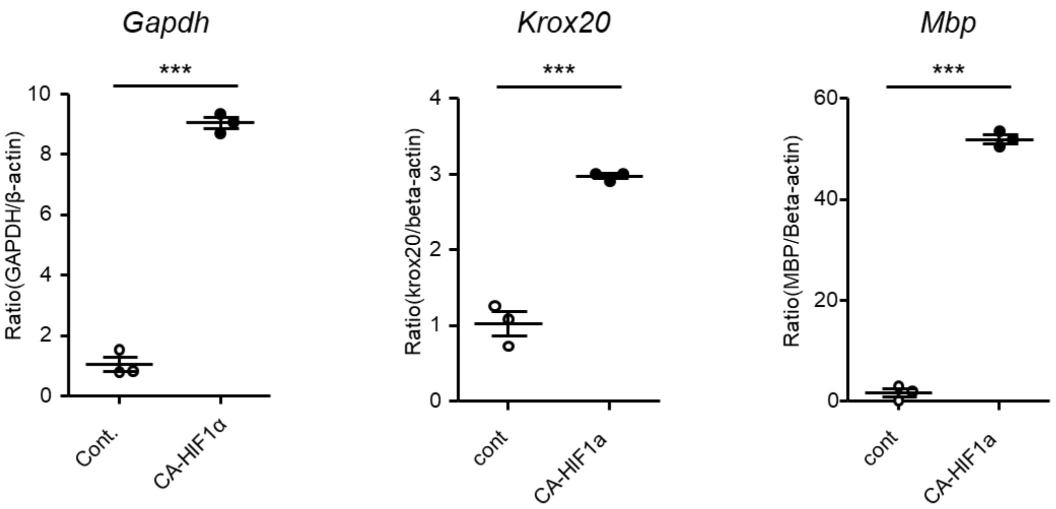

Graph depicting quantitative RT-PCR analysis of the indicated genes in primary cultured Schwann cells transfected with the respective plasmids. Gene expression levels were normalized to β-actin and are shown relative to control cells subjected to electroporation alone. (n=3; ***P<0.001 by unpaired Student's t-test; mean ± SEM.)

Supplementary Figure 4

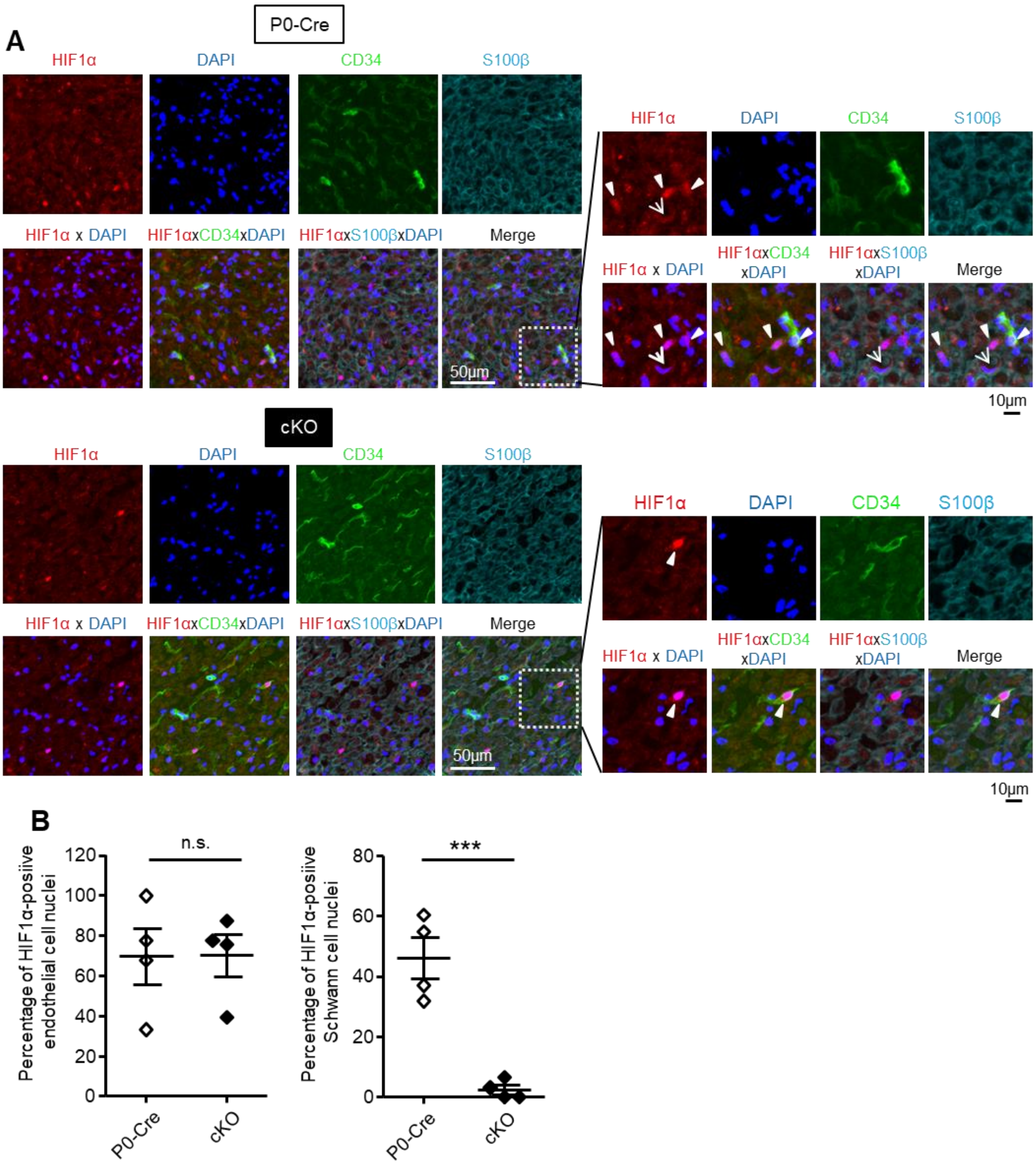

A) Representative immunofluorescence micrograph depicting the cellular localization of HIF1α in a cross-section of the injured sciatic nerve, 5 days after nerve crush. Red: HIF1α; Green: CD34 (vascular endothelial cells); Light blue: S100β (Schwann cells); Blue: DAPI. Scale bar: 50 µm. A magnified view of the region outlined by the white box is shown on the right, scale bar: 10 µm. White arrowheads denote nuclei of HIF1α-positive vascular endothelial cells, while white arrows indicate nuclei of HIF1α-positive Schwann cells.

B) Quantification of the proportion of HIF1α-positive endothelial cell nuclei or Schwann cell nuclei in

sciatic nerve cross-sections, shown in (A), from P0-Cre: HIF1α$^{flox/flox}$ (cKO) and P0-Cre mice. n =4. ***P<0.001 by unpaired Student's t-test; mean ± SEM.
